# Impact of glucose-6-phosphate dehydrogenase deficiency on dengue infection in Myanmar children

**DOI:** 10.1371/journal.pone.0209204

**Published:** 2019-01-02

**Authors:** Win Lai May, Myat Phone Kyaw, Stuart D. Blacksell, Sasithon Pukrittayakamee, Kesinee Chotivanich, Borimas Hanboonkunupakarn, Khin Nyo Thein, Chae Seung Lim, Janjira Thaipadungpanit, Thomas Althaus, Podjanee Jittamala

**Affiliations:** 1 Department of Clinical Tropical Medicine, Faculty of Tropical Medicine, Mahidol University, Bangkok, Thailand; 2 Department of Medical Research, Yangon, Myanmar; 3 Mahidol-Oxford Tropical Medicine Research Unit, Faculty of Tropical Medicine, Mahidol University, Bangkok, Thailand; 4 Centre for Tropical Medicine and Global Health, Nuffield Department of Medicine, University of Oxford, Oxford, United Kingdom; 5 Department of Paediatrics, University of Medicine 2, Yangon, Myanmar; 6 Korea University, Guro Hospital, Seoul, Korea; 7 Department of Tropical Hygiene, Faculty of Tropical Medicine, Mahidol University, Bangkok, Thailand; Faculty of Science, Ain Shams University (ASU), EGYPT

## Abstract

Glucose-6-phosphate dehydrogenase (G6PD) deficiency may affect the clinical presentation of dengue due to the altered redox state in immune cells. We aimed to determine the association between G6PD deficiency and severity of dengue infection in paediatric patients in Myanmar. A cross-sectional study was conducted among paediatric patients aged 2–13 years with dengue in Yankin Children Hospital, Myanmar. One hundred and ninety-six patients positive for dengue infection, as determined via PCR or ELISA, were enrolled. Dengue severity was determined according to the 2009 WHO classification guidelines. Spectrophotometric assays determined G6PD levels. The adjusted median G6PD value of males in the study population was used to define various cut-off points according to the WHO classification guidelines. G6PD genotyping for Mahidol, Kaiping and Mediterranean mutations was performed for 128 out of 196 samples by real-time multiplex PCR. 51 of 196 (26.0%) patients had severe dengue. The prevalence of G6PD phenotype deficiency (< 60% activity) in paediatric patients was 14.8% (29/196), specifically, 13.6% (14/103) in males and 16.2% (15/93) in females. Severe deficiency (< 10% activity) accounted for 7.1% (14/196) of our cohort, occurring 11.7% (12/103) in males and 2.2% (2/93) in females. Among 128 samples genotyped, the G6PD gene mutations were detected in 19.5% (25/128) of patients, with 20.3% (13/ 64) in males and 18.8% (12/64) in females. The G6PD Mahidol mutation was 96.0% (24/25) while the G6PD Kaiping mutation was 4.0% (1/25). Severe dengue was not associated with G6PD enzyme deficiency or presence of the G6PD gene mutation. Thus, no association between G6PD deficiency and dengue severity could be detected.

**Trial registration**: The study was registered following the WHO International Clinical Trials Registry Platform (WHO-ICTRP) on Thai Clinical Trials Registry (TCTR) website, registration number # TCTR20180720001

## Introduction

Glucose-6-phosphate dehydrogenase (G6PD) is an enzyme presenting in the cytoplasm of human cells that participates in the pentose phosphate pathway and supplies reducing energy by maintaining levels of the co-enzyme nicotinamide adenine dinucleotide phosphate (NADPH) [1]. G6PD deficiency is an X-linked inheritance. G6PD gene consists of 13 exons with approximately 18kb and is situated on the distal long arm of X chromosome (Xq28). About 180 mutations of G6PD gene have been reported resulting in protein variants with different levels of enzyme activity. NADPH is essential for both oxidant and antioxidant systems of cells. In the antioxidant pathway, NADPH maintains the reduced form of glutathione to protect cells from oxidative damage [2]. This reduction in cells causes accumulation of redox oxidative species (ROS) and leads to senescence [1] and haemolysis in red blood cell [3]. On the other hand, NADPH is also involved in the oxidant pathway to produce ROS. Although the overproduction of these ROS may adversely affect cell function, cells of immune system also need these reactive species to kill invading organisms. Phagocytes of the immune system need these reactive species to kill invading pathogens as part of the innate immune system. In cases of severe G6PD deficiency, the lack of oxidative metabolism can cause a reduction in oxygen-dependent phagocytosis as observed in chronic granulomatous disease [4] and allows for viral replication [5–8].

Glucose-6-phosphate dehydrogenase (G6PD) deficiency affects more than 400 million people all over the world [9] and prevalence is approximately 35% in Africa and ranges from 6.0 to 10.8% in Southeast Asia [10]. In Myanmar, the prevalence is 11.1% and 4.2% in adult males and females respectively [11], while it is 15.0% and 2.1% in healthy children males and females respectively [12]. To note, 11.8% of males and 21.0% of females possess the G6PD mutation [13] with the G6PD Mahidol variant occurring in 91.3% of children in Myanmar [14].

Dengue virus infection is one of the leading causes of morbidity and mortality in children living in tropical and sub-tropical regions [15]. According to WHO, approximately 390 million people worldwide experience a dengue infection annually [16]. In Southeast Asia, dengue leads to over 5,900 deaths annually [17]. In Myanmar, all four dengue serotypes are known to co-circulate, and children are at the highest risk for infection [18]. The reported case fatality rate was 7 per 1,000 dengue cases in 2014 [19].

Most dengue patients present with undifferentiated febrile illness that some may progress to life threatening disease [20]. How patients’ genetic background affects the development of severe infection has become an area of interest. G6PD deficiency is one of the reported genetic variants associated with infections [8, 21]. *In vitro* studies reported that monocytes from G6PD-deficient individuals had increased susceptibility to dengue virus serotype 2 infections along with higher viral replication [5, 6]. Whether higher replication of dengue virus in G6PD-deficient individuals increases the likelihood of disease severity remains unknown. Herein, we investigated the association between G6PD deficiency and severity of dengue infection in paediatric patients in Myanmar.

## Materials and methods

### Patient recruitment and sample collection

A cross-sectional prospective study was conducted in Yankin Children Hospital, Yangon from August 2015 to August 2016. Paediatric patients (age range: 2–13 years) with a fever for 1–7 days and clinically suspected of having dengue fever were screened. Written informed consent and verbal assent were taken from caregivers and children, respectively. Three millilitres of blood were taken, and sera were tested for the NS1 antigen as well as for IgM and IgG antibodies using the SD BIOLINE Dengue Duo rapid diagnostic test (RDT) (Standard Diagnostics, Korea). Patients with a positive NS1 antigen and/or dengue IgM result were enrolled. Patients who had a diagnosis other than dengue, such as co-infections and immunocompromised status (e.g., steroid use, chemotherapy, and/or positive HIV status) were excluded. Patients were followed 1–3 weeks after admission for convalescent serum sample collection. Complete blood count (CBC), reticulocyte count (RC) and G6PD activity were measured within 24 hours after collection of acute sera. G6PD activity was assessed via spectrophotometry (Randox Laboratory, UK). The adjusted median value of G6PD activity for males in our cohort was used to define various cut-off points [22]. Dengue severity was classified according to the 2009 WHO classification guidelines [23]. Both acute and convalescent samples were stored at -80°C prior to further testing.

The G6PD activity, CBC and RC were re-checked at least one month after the first sample collection for patients at risk of being misdiagnosed for G6PD deficiency based on analyses of the first blood sample collected, such as patients with RC values > 2%, those who suffered or recovered from acute haemolytic anaemia, and those who had a blood transfusion before the first sample collection.

The study protocol was approved by the Ethics Review Committee of the Department of Medical Research, Myanmar (approval number 64/Ethics 2015) and the Ethics Committee of the Faculty of Tropical Medicine, Mahidol University, Thailand (approval number was 2016-0808-01). The study was also registered following the WHO International Clinical Trials Registry Platform (WHO-ICTRP) on Thai Clinical Trials Registry (TCTR) website, number # TCTR20180720001.

The data that support the findings of this study are available in protocols.io with the https://dx.doi.org/10.17504/protocols.io.u9fez3n.

### Reference testing

#### Dengue testing

Dengue infection was confirmed by quantitative real-time RT-PCR and NS1 antigen, IgM and IgG ELISAs at the Mahidol Oxford Research Unit, Thailand. Dengue virus NS1 ELISA (Cat no. 11EK50, Standard Diagnostics Inc., South Korea), dengue virus IgM ELISA (Cat no. 11EK20, Standard Diagnostics Inc., South Korea), and dengue virus IgG ELISA (Cat no. 11EK10, Standard Diagnostics Inc., South Korea) were all performed according to the manufacturer’s instructions.

#### Dengue, zika and chikungunya virus detection using a reverse transcriptase PCR assay

RNA was isolated from acute patient serum samples (140 μL) using the QIAamp Viral RNA Mini Kit (Qiagen, Germany). RNA extractions were performed according to the manufacturer’s protocol. RNA was stored at -80°C until further use. One-step reverse transcriptase (RT) PCR was performed to detect dengue, zika and chikungunya viruses in a single assay using ZDC Multiples RT-PCR Assay (Bio-Rad, USA). Assays were performed according to the manufacturer’s protocol, with an additional RNase P3 primer mix as an internal extraction control. For each RT-PCR assay, a positive control, provided from the kit; negative control (healthy donor sera); and no template control were included. All RNA samples were performed in duplicate. A positive PCR was defined as when one or both duplicates had a signal above a fixed threshold of 200 for all the respective targets: FAM for zika virus, HEX for chikungunya virus, and Texas Red for dengue virus.

#### G6PD activity

Sample was stored at 4–8°C, then transported to Department of Medical Research (DMR) laboratory in Yangon Myanmar and processed within 24 hours. Spectrophotometry was conducted using Humalyser 3000 spectrophotometer (Medsource Ozone Biomedicals, Delhi, India) and kits from Randox (Cat no PD 410, Randox Laboratories Ltd., Crumlin, UK). The procedure provided in the manual with the kit was followed. Normal and deficient G6PD controls (Cat no PD 2617 for deficient and Cat no PD 2618 for normal, Randox Laboratories Ltd., Crumlin, UK) were used. The results were considered valid if the measured activities of the controls were within the reference range. Enzyme activity was determined by the spectrophotometer set at 37°C to measure the rate of absorbance at 340 nm under ultraviolet light. Enzyme activity was calculated and adjusted for the Hb concentration value from complete blood count (CBC) recorded at the time of sample collection. The adjusted median value of G6PD activity for males in our cohort was used to define various cut-off points [22].

#### G6PD genotyping by real-time PCR

DNA samples were extracted from 200 μL of whole blood using the QIAamp DNA Blood Mini Kit (Qiagen, Germany) according to the manufacturer’s recommendation. Three G6PD variants (G6PD Mahidol c.487G>A; G6PD Kaiping c.1388G>A; and G6PD Mediterranean c.563C>T) previously reported in G6PD-deficient individuals in Myanmar were detected by real-time PCR with the BIO-RAD CFX 96 Real-Time System and C1000 Thermal Cycler (Bio-Rad, USA). Genotyping PCR, with melting curve analysis using dual-labelled, self-quenched probes, was performed using 25 μL reactions, containing 12.5 μL of iQ Multiplex Power Mix, 0.75 μL each of forward and reverse primers, 0.5 μl each of SNP and WT probes, 9 μL sterile distilled water, and 1 μL genomic DNA. The amplification conditions were 3 minutes at 95°C for iQ Multiplex Power Mix activity, followed by 40 cycles of 15 seconds at 95°C for denaturation, and 1 min at 70°C for annealing and extension for G6PD genotyping. The samples were run together with the plasmid control for each targeted mutation; three separate PCR reactions were run in every sample for the three different mutations. Fluorescence and Ct values using the CFX Manager software (Bio-Rad, USA) were used to determine genotypes. Each sample’s result was verified by examining the PCR curve generated to eliminate false-positive results due to aberrant light emission (S1 Table).

### Case definitions

#### Acute dengue infection

Laboratory confirmed acute dengue infection was defined by a positive qRT-PCR, NS1 antigen (Ag) ELISA, or IgM ELISA result. Primary or secondary dengue infections were defined according to a negative or positive IgG ELISA result in the febrile phase (≤ 7 days), respectively [24].

#### Dengue severity

The severity of dengue infection was classified according to the 2009 WHO guideline [23]. Dengue patients were classified as non-severe or severe based on clinical and laboratory parameters. Patients with non-severe dengue were classified into two groups based on the absence or presence of warning signs. Non-severe dengue without warning signs was defined if the patient had fever with 2 of these following criteria: nausea/vomiting, rash, aches and pains, tourniquet test positive, leucopoenia without presentation of any warning signs. Warning signs included abdominal pain or tenderness, persistent vomiting, clinical fluid accumulation, mucosal bleed, lethargy/restlessness, liver enlargement > 2cm and increase in haematocrit (≥ 44%) concurrent with rapid decrease in platelet count (<100 × 10^3^ per μl). Severe dengue was defined as: dengue shock syndrome (DSS), fluid accumulation with respiratory distress, and/or severe bleeding that necessitated blood transfusion or involved an organ [23]. DSS was diagnosed by the presence of hypotension or narrow pulse pressure (< 20 mmHg) [25].

#### G6PD deficiency

The WHO classification is based on the G6PD activity expressed as a percentage of the median G6PD value of a normal male population. Class I-V are defined as: class I < 1% (associated with chronic non-spherocytic haemolytic anaemia), class II 1-< 10%, class III 10-<60%, class IV 60–150%, and class V > 150%. In this study, G6PD deficiency was classified as severe, moderate, or normal if enzyme activity was < 10%, 10–60%, or > 60%, respectively [26]. The adjusted median enzyme activity in males was determined as 100% activity of the study population [22].

### Statistical analysis

Only patients with laboratory confirmed acute dengue infection were included in the statistical analysis. Data were evaluated using descriptive statistics, medians (interquartile ranges) for continuous variables and frequencies and percentages for categorical variables. Chi-squared test or Fisher’s exact test (for categorical variables) and Mann–Whitney U test (for continuous variables) were used for comparative analyses between severe and non-severe dengue infections and between patients with or without G6PD deficiency. Associations between G6PD status and dengue severity were evaluated using the binary logistic regression test with 95% confidence interval. Data were analysed using IBM SPSS statistics (IBM, Armonk, NY, USA) data manager software package.

## Results

### Patient recruitment

**Fig 1:**
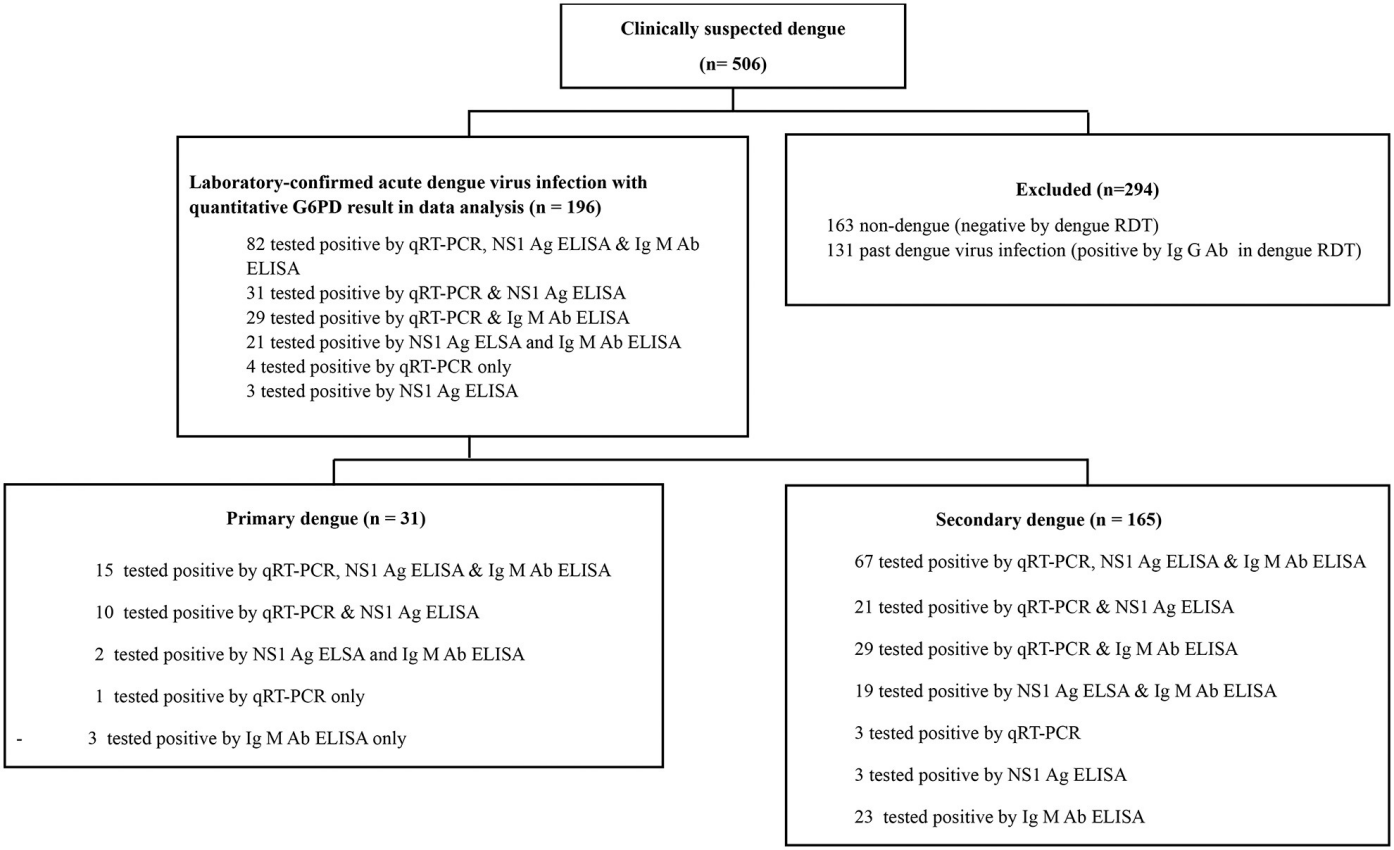
Study flowchart.

In total, 506 patients with clinically suspected dengue infection were screened by RDT. In our study, 294 (58.0%) patients were excluded (163 non-dengue and 131 with past dengue infection), and 212 patients were enrolled in the study (defined as positive NS1 Ag and/or IgM result) as indicated on Fig 1. Out of 212 enrolled patients, 16 were excluded (2 did not have sufficient blood volume, 11 had a negative dengue result, and 3 lacked quantitative G6PD results). In total, 196 patients with laboratory confirmed acute dengue infection and with quantitative G6PD results were included in the analysis (Fig 1).

### Dengue severity

Among the 196 patients with a confirmed acute dengue infection, 51 (26.0%) were classified as severe dengue while 145 (74.0%) were classified as non-severe dengue. Among the 145 patients with non-severe dengue, 115 (79.3%) presented with warning signs and 30 (20.7%) were without. All patients with severe dengue presented with dengue shock syndrome (DSS). Among these DSS patients, one patient also had fluid overload with respiratory distress, and one patient had severe bleeding that necessitated blood transfusion. Ten out of fifty-one (20.0%) of children with severe dengue were admitted to the ICU, and 11 required a blood transfusion. Patients with severe dengue were significantly younger (p = 0.001) and were hospitalised for a longer time (p <0.001) compared to non-severe dengue patients. The majority of patients were classified as secondary dengue (84.2%, 165/196), and only 15.8% (31/196) were classified as primary dengue. Severe dengue was significantly higher in secondary dengue patients compared with the non-severe group (p = 0.007).

Nausea and vomiting were more frequent in patients with severe dengue (p = 0.001). However, a positive tourniquet test was less frequent in children with severe dengue (p = 0.007). Other clinical warning signs, including tachycardia and hypotension, were significantly detected in patients with severe dengue. (p <0.001). Patients with severe dengue also had significantly higher haematocrit, higher leukocyte counts, and lower platelet counts than non-severe dengue patients (Table 1).

**Table 1:** Demographics, clinical and haematological characteristics of confirmed dengue paediatric patients in Yankin Children Hospital, Yangon, Myanmar.

Characteristics	Severe-dengue Number (%) (n = 51)	Non-severe dengue Number (%) (n = 145)	p-value
**Baseline Characteristics**			
Male	23 (45.1)	80 (55.2)	0.215
Age (year),median (IQR)*	6.3 (5–9)	8 (5.9–10)	**0.011**
Day of fever on admission (day), median (IQR)*	4 (4–5)	4 (3–5)	0.250
Secondary dengue	49 (96.1)	116 (80.0)	**0.007**
ICU	10 (19.6)	0 (0)	**<0.001**
Blood transfusion	11 (21.6)	0 (0)	**<0.001**
Hospital stay (day),median (IQR)*	3 (2–3)	2 (2–3)	**<0.001**
**Non-specific clinical presentations**			
Headache	17 (33.3)	62 (42.8)	0.238
Retro orbital pain	3 (5.9)	16 (11)	0.411
Red eyes	5 (9.8)	19 (13.1)	0.536
Sore throat	6 (14.5)	21 (11.8)	0.628
Ecchymosis/bruising	2 (3.9)	1 (0.7)	0.166
Spontaneous petechiae	6 (11.8)	18 (12.4)	0.903
Nausea/vomiting	43 (84.3)	85(58.6)	**0.001**
Rash	4 (7.8)	26 (17.9)	0.085
Aches and pain	24 (47.1)	68 (46.9)	0.984
Tourniquet test positive	24/32 (75)	129/139 (92.8)	**0.007**
**Warning signs**			
Abdominal pain/tenderness	35 (68.6)	53 (36.6)	**<0.001**
Clinical fluid accumulation	6 (11.8)	1 (0.7)	**0.001**
Mucosal bleed	14 (27.5)	46 (31.7)	0.569
Lethargy/restlessness	48 (94.1)	49 (33.8)	**<0.001**
Liver enlargement >2 cm	46 (90.2)	70 (48.3)	**<0.001**
**Vital signs**			
Highest pulse rate (beat/min.),median (IQR)*	120 (110–130)	102 (93–120)	**<0.001**
Lowest systolic blood pressure (mmHg),median (IQR)*	80 (80–90)	90 (90–100)	**<0.001**
**Haematological parameters**			
Highest haematocrit (%), median (IQR)*	45 (41–47)	37.6 (35–41)	**<0.001**
Leukocyte count (10^3^ cells per μl),median (IQR)*	5.4 (3.9–8.7)	3.7 (2.9–5.3)	<0.001*
Lowest platelet count, (10^3^ per μl), median (IQR)*	52 (28–74)	120 (80–173)	**<0.001**

* IQR, interquartile range

### G6PD status and genotype

The median G6PD activity of 196 dengue-confirmed patients was 5.4 U/g Hb (0.1–11.5). The adjusted male median value of G6PD activity was determined by excluding 12 male samples with ≤ 10% activity from the derived value [22]. The adjusted male median was 5.7 U/g Hb (1.2–11.5) (Table 2). Based on this value, G6PD activity < 0.057 U/g Hb was defined as Class I, activity between 0.057–0.57 U/g Hb was Class II, 0.57–3.42 U/g Hb was Class III, 3.42–8.55 U/g Hb was Class IV, and > 8.55 U/g Hb was Class V for this population (Table 3).

**Table 2:** Reference values for G6PD activity among confirmed dengue paediatric patients.

Reference values (U/g Hb)	Total (n = 196)	Female (n = 93)	Male (n = 103)	Adjusted Male* (n = 91)
Median (IQR)^¥^	5.4 (0.1–11.5)	5.3 (0.2–9.7)	5.4 (0.1–11.5)	5.7 (1.2–11.5)

^¥^ Interquartile range

* These values exclude males with severe G6PD deficiency defined as <10% of the G6PD median value for all males in the study population.

**Table 3:** Prevalence of G6PD among confirmed dengue paediatric patients according to WHO classification.

	WHO classification (% of G6PD based on male adjusted value) (U/g Hb)				
	Class I (<1%) <0.057	Class II (1–10%) 0.057–0.57	Class III (10–60%) 0.57–3.42	Class IV (60–150%) 3.42–8.55	Class V (>150%) >8.55
Male, n (%) (n = 103)	0	12 (11.7)	2 (1.9)	83 (80.6)	6 (5.8)
Female, n (%) (n = 93)	0	2 (2.2)	13 (14.0)	75 (80.6)	3 (3.2)
**Total**, n (%)	0	14 (7.1)	15 (7.7)	158 (80.6)	9 (4.6)

The prevalence of G6PD phenotypic deficiency, based on these cut-off reference values revealed that 14/196 (7.1%), including 12 (11.7%) males and 2 (2.2%) females were in Class II (severe G6PD deficiency) and 15/196 (7.7%), including 2 (1.9%) males and 13 (14.0%) females were in Class III (moderate G6PD deficiency) (Table 3).

Real-time PCR for three known single nucleotide polymorphisms (SNPs) in the G6PD gene in Myanmar, Mahidol, Kaiping and Mediterranean, was performed in 128 of 196 dengue-confirmed samples (64 males and 64 females). Among them, 25/128 (19.5%) had recognised SNPs, (13/64 (20.3%) were males while 12/64 (18.8%) were females). Twenty-four patients had the Mahidol mutation (96%), and one female (4%) had the Kaiping mutation. The Mediterranean genotype was not detected. Samples from of 28/29 (96.5%) (14/ 14 males, 14/15 females) patients with G6PD deficient phenotypes were included in this genotypic study. Among them, only 20/ 28 (71.4%) had mutations, while 9/ 28 (32.1%) (2 male and 7 female) did not have mutation. Among the samples from patients without G6PD phenotype deficient, 6 / 100 (6%) samples (1 male and 5 female) had mutations (Table 4).

**Table 4:** G6PD genotyping in paediatric patients with confirmed dengue.

G6PD activity	Gender	Total sample (N)	Sample for Genotyping (n_1_/N (%))	Sample with mutation (n_2_/n_1_ (%))	Sample without mutation (n_3_/n_1_ (%))
>150% activity	Male	6	2/6 (33.3)	0/2 (0.0)	2/2 (100)
	Female	3	2/3 (66.7)	0/3 (0.0)	3/3 (100)
60%-150% activity	Male	83	48/83 (57.8)	1/48 (2.0)	47/48 (97.9)
	Female	75	48/75 (64.0)	5/48 (10.4)	43/48 (89.6)
<60% activity	Male	14	14/14 (100)	12/14 (85.7)	2/14 (14.3)
	Female	15	14/15 (93.3)	7/14 (50.0)	7/14 (50.0)
**Total**		196	128/196 (65.3)	25/128 (19.5)	103/128 (80.5)

**Fig 2:**
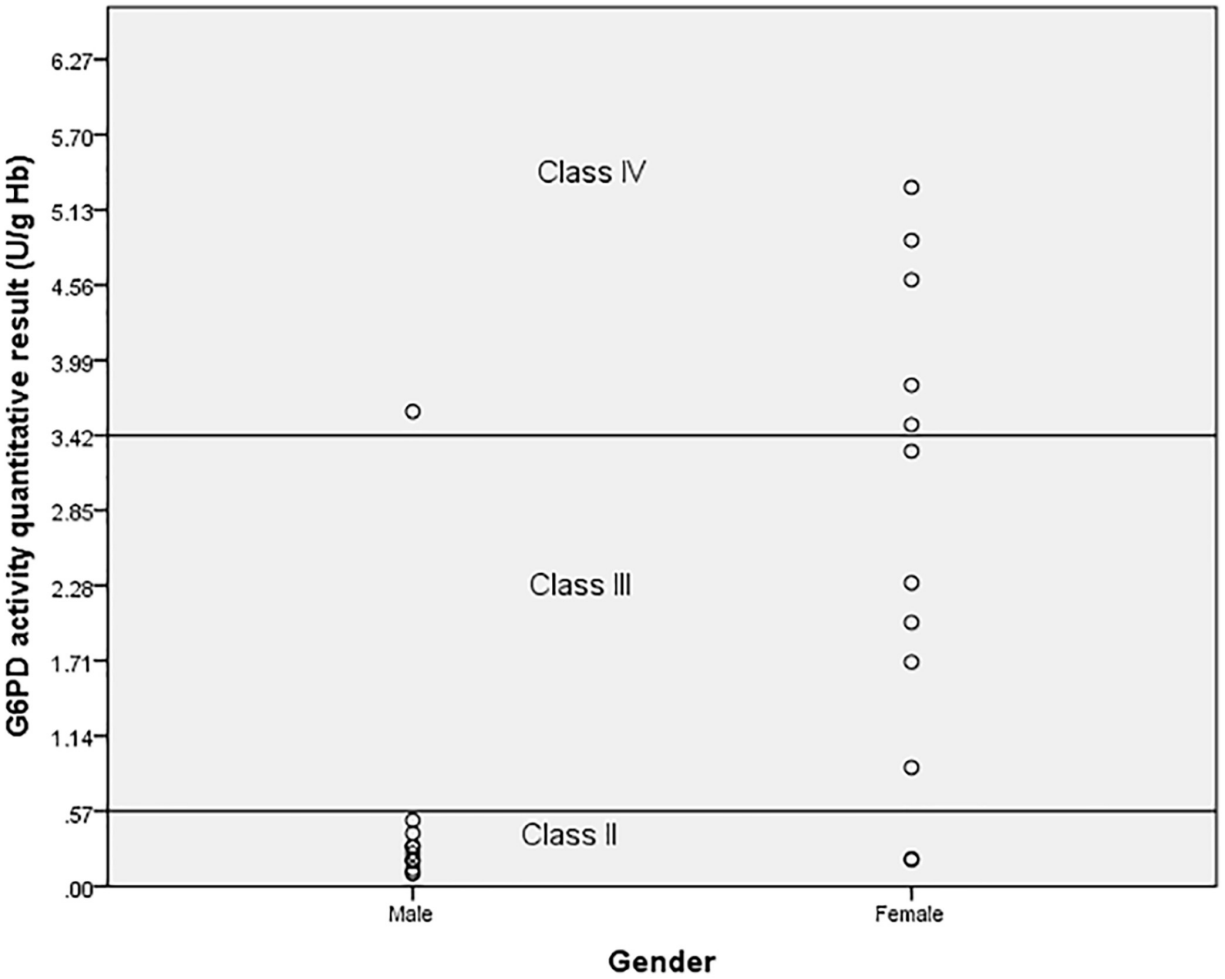
Phenotype distribution among males and females with G6PD gene mutation according to WHO classification.

Among the 25 patients with a G6PD mutation, 14/25 (56.0%) were classified as WHO class II and among them 13/14 (92.8%) were heterozygous male and one homozygous female. There was one male with the Mahidol mutation in WHO Class IV. This patient had a reticulocyte count < 2% while testing for G6PD activity. Females with genotypic mutations have varying degrees of the G6PD deficient phenotype being classified as WHO class II to WHO class IV (Fig 2).

### Association of dengue severity and G6PD status

Severe dengue was not associated with a G6PD deficiency phenotype nor genotype variants whether we used a cut off of < 30% (i.e. only including hemizygous males and homozygous females) or a cut off of < 60%, corresponding to classes I to III of the WHO classification (Table 5).

**Table 5:** G6PD status related to severe and non-severe dengue among confirmed dengue paediatric patients.

G6PD status	Severe dengue n (%)	Non-severe dengue n (%)	Logistic regression Crude OR (95% CI)	p-value
Enzyme activity <30% (deficient) (n = 18)	1 (2.0)	17 (11.7)	0.15 (0.02–1.16)	0.069
Enzyme activity ≥30% (non-deficient), (n = 178)	50 (98.0)	128 (88.3)		
Enzyme activity <60% (deficient) (n = 29)	5 (9.8)	24 (16.6)	0.5 (0.2–1.5)	0.249
Enzyme activity ≥60% (non-deficient), (n = 167)	46 (90.2)	121 (83.4)		
Gene mutation present (n = 25)	6 (19.4)	19 (19.6)	0.9 (0.3–2.7)	0.977
Gene mutation absent (n = 103)	25 (80.6)	78 (80.4)		

Severe dengue was diagnosed in 5/29 (17.2%) children with < 60% enzyme activity and in 46/167 (28%) children with G6PD levels > 60% (p = 0.249). In addition, 6/25 (24%) study participants had a G6PD SNP, and 25/103 (24%) did not have a detectable SNP and severe dengue (p = 0.977).

## Discussion

Herein, we investigated if there is an association between dengue fever and G6PD deficiency in children in Myanmar. The majority of children in this study, 84.2%, had secondary dengue infection possibly due to the four dengue serotypes circulating in this endemic area, which leads to subsequent infections [19]. A quarter of the children in the present study had a severe dengue fever, all of whom presented with DSS. The frequency of secondary dengue infection was significantly higher in the severe dengue than in the non-severe dengue group, which is consistent with a meta-analysis of 40 studies in Asia that described the association between DSS and secondary dengue infection [27].

No association between severity of dengue infection and G6PD enzyme deficiency or G6PD mutation was detected. The result of this study is consistent with two earlier studies conducted in Thailand [28, 29]. Tanphaichitr and colleagues did not demonstrate a correlation between G6PD deficiency and dengue severity in 89 male paediatric patients (age range: 1–13 years) with dengue haemorrhagic fever age. In this study, 17 out of 72 patients had G6PD deficiency [28]. Seridhoranakul *et al*. also did not demonstrate a significant relationship between G6PD deficiency and DHF in a study with 80 DHF patients, in which 9 patients were G6PD-deficient, and 131 controls, in which 13 patients were G6PD-deficient [29]. Tanphaichitr *et al*. reported a higher prevalence of G6PD deficiency in male DHF patients, which was 19.1% compared to 12% in Bangkok, and suggested that G6PD-deficient males may suffer more from DHF [28]. The prevalence of G6PD deficiency in paediatric patients with dengue infection in the present study was lower than the prevalence of G6PD deficiency in children visiting the Emergency Department of Yankin Children Hospital, ages: 1 month to 12 years old, who were screened for dengue via RDT (14.8% vs. 18.5%, respectively). In this study, G6PD deficiency was diagnosed if enzyme activity was < 60% of the adjusted median value, which was slightly lower than the adjusted median value in our study (3.24 U/g Hb vs. 3.42 U/g Hb) [30]. When comparing the results of these two studies, conducted in the same setting, G6PD-deficient children may not be more susceptible to dengue infection compared to children with normal G6PD activity.

Two *in vitro* studies demonstrated that human monocytes from G6PD-deficient individuals had higher replication of dengue virus serotype 2 [5, 6], and these studies suggested that the likelihood of severe dengue was likely to increase in G6PD-deficient individuals. The findings from our study did not support this hypothesis. However, viral load was not assessed in our study, thus, the association of viral load or viral replication to clinical outcomes was beyond the scope of this study. The different dengue serotype also affects clinical outcomes. Although dengue serotypes were not assessed in this study, previous studies described that the prevalent serotype among patients with DHF in Myanmar in recent years was dengue serotype 1 [31, 32]. Additionally, all four serotypes participated in the 2015 outbreak [19]. The association between G6PD deficiency, viral load and different dengue serotypes warrant future studies.

More than 180 different mutations in the G6PD gene have been identified, and these variants have different impacts on enzyme activity [33], which might result in different clinical manifestations. One study reported that patients with the G6PD Mediterranean mutation showed a more severe clinical course during bacterial infection compared to patients with wild type G6PD [34].

Most patients with G6PD mutation possessed the Mahidol variant, which is consistent with previous studies in Myanmar [10, 14]. Genotypic mutation was not detected in 2 male and 7 female patients with phenotypic deficiency and may have been due to the limited number of genotypes that we sought; previous research in this region has identified other uncommon G6PD mutations for instance G6PD Union, Coimbra and Canton [14, 35, 36]. Bancone et al reported this molecular heterogeneity among this ethnicity along Thai-Myanmar border [35]. Six patients (1 male and 5 females) who had genotypic mutation had normal G6PD activities; all had reticulocyte count < 2%, excluding a false negative phenotype due to haemolysis. G6PD Mahidol is considered a moderately severe variant with varying G6PD activities which classifies it as WHO class II/III; almost all of male patients with Mahidol variant had enzyme activities < 10% (i.e. were class II). This finding is consistent with results of another study from the Thai-Myanmar border [35]. A significant association between G6PD mutation, in predominantly the Mahidol variant, and dengue infection was not found. Whether G6PD genotypes other than Mahidol could affect dengue severity cannot be excluded based on our current study. Thus, the effect of other G6PD gene mutations on dengue infection is unknown, and complementary studies should be performed in different populations.

In this study, the demographic, clinical and haematological parameters of patients with severe and non-severe dengue were compared, and we found that young age is a risk factor significantly associated with severe dengue infection. Data collected from 2011 to 2015 in Myanmar also reported that the highest case fatality rate was seen in the infantile age group [19].

The clinical parameters of warning signs were more frequent in severe dengue patients, which was in agreement with previous studies; hepatomegaly was a risk factor of DSS or severe dengue infection [37–42], abdominal pain was a prognostic factor for severe dengue [43, 44], and lethargy was the best clinical sign to identify patients who may progress to severe dengue [45]. Although there were no patients with persistent vomiting, those who had a history of vomiting were frequently more present in the severe dengue patient group. Other studies reported that persistent vomiting was one of the factors associated with DSS or high mortality [27, 37]. In the 2009 WHO classification for dengue, high haematocrit ≥ 20% from baseline, concurrent with a rapidly decreasing platelet count, is listed as a warning sign [23]. In this study, patients with severe dengue had significantly higher haematocrit and lower platelet count than those with non-severe dengue. The findings in this study also reinforced the applicability of the warning signs outlined in the 2009 WHO guidelines to detect severe dengue infection in children in Myanmar.

The strength of this study was its prospective design. All acute dengue cases were confirmed by real-time PCR for the virus and ELISA assays, and G6PD deficiency was confirmed by a gold standard quantitative spectrophotometric assay. Although the cut-off level of G6PD activity did not represent all children in Myanmar, it did reflect the G6PD activity of children with dengue infection seen at the Yankin Children Hospital.

There were limitations in our study. For example, dengue viral load and serotypes were not analysed; therefore, we were not able to detect the relationship among viral load, serotypes, dengue severity and G6PD deficiency. Additionally, G6PD mutations were not assessed in all the participants, and the impact of rare G6PD variants previously reported in Myanmar population could not be analysed, which may have impacted our statistical analysis. The parameters, e.g., chest X-rays, liver function tests and ultrasounds of chest and abdomen that may affect dengue severity classification in terms of major organ involvement or evidence of plasma leakage were not collected, and these factors may have affected our study’s outcomes.

## Conclusion

We did not find an association between G6PD deficiency and dengue severity. Most of our patients had G6PD Mahidol, the most common variant in Myanmar. This study proposes activity cut–off points of enzyme activity according to the WHO classification, as well as the prevalence of G6PD deficient and the predominant genetic mutation among paediatric patients. This study reconfirms the usefulness of the WHO 2009 dengue classification in paediatric dengue patients in Myanmar. More research is needed to explore other factors related to dengue severity such as dengue virus viral load, dengue virus DEN serotypes and genotypes.

## Supporting information

S1 Table Forward, reverse primers, SNPs and WT probes used in the study. (DOCX)
