# Supplementary material for: Impact of glucose-6-phosphate dehydrogenase deficiency on dengue infection in Myanmar children
Source: PLoS One. 2019 Jan 2;14(1):e0209204. doi: 10.1371/journal.pone.0209204 (PMC6314580; doi:10.1371/journal.pone.0209204)
Supplement: S1 Table — (DOCX) [file pone.0209204.s001.docx]

**S1.** Forward, reverse primers, SNPs and WT probes used in the study

| **Forward and Reverse Primers**  Mahidol F- AGAGGGGTTCAAGGGGGTAA, Mahidol R- CCGAAGGGCTTCTCCA CGAT,  Kaiping F- CTATGGGGTGGCCTTTGCC, Kaiping R- CCCTTTCCTCACCTGCC ATA,  Mediterranean F- ACCGCATCATCGTGGAGAAG, Mediterranean R- GGTAGTGGTCGATGCGGTAG  **SNP and WT probes**  Mahidol SNP- [5FAM]TGCGGTTCCAGCTTCTGCTGGGAG[3BHQ1], Mahidol WT-[5CY5]TGCGGTTCCAGCCTCTGCTGGGAG[3BHQ2]  Kaiping SNP-[5FAM]CCGTGAGGCCTGGCATATTTTCACCC[3BHQ1], Kaiping WT- [5CY5]CCGTGAGGCCTGGCGTATTTTCACCC[3BHQ2]  Mediterranean SNP- [5FAM]CCACATCTTCTCCCTGTTCCGTGAGG[3BHQ1], Mediterranean WT- [5CY5]CCACATCTCCTCCCTGTTCCGTGAGG[3BHQ2]) |
| --- |
